# Both cold and sub-zero acclimation induce cell wall modification and changes in the extracellular proteome in *Arabidopsis thaliana*

**DOI:** 10.1038/s41598-019-38688-3

**Published:** 2019-02-19

**Authors:** Daisuke Takahashi, Michal Gorka, Alexander Erban, Alexander Graf, Joachim Kopka, Ellen Zuther, Dirk K. Hincha

**Affiliations:** Max-Planck-Institut für Molekulare Pflanzenphysiologie, Am Mühlenberg 1, D-14476 Potsdam, Germany

## Abstract

Cold acclimation (CA) leads to increased plant freezing tolerance during exposure to low, non-freezing temperatures as a result of many physiological, biochemical and molecular changes that have been extensively investigated. In addition, many plant species, such as *Arabidopsis thaliana*, respond to a subsequent exposure to mild, non-damaging freezing temperatures with an additional increase in freezing tolerance referred to as sub-zero acclimation (SZA). There is comparatively little information available about the molecular basis of SZA. However, previous transcriptomic studies indicated that cell wall modification may play an important role during SZA. Here we show that CA and SZA are accompanied by extensive changes in cell wall amount, composition and structure. While CA leads to a significant increase in cell wall amount, the relative proportions of pectin, hemicellulose and cellulose remained unaltered during both CA and SZA. However, both treatments resulted in more subtle changes in structure as determined by infrared spectroscopy and monosaccharide composition as determined by gas chromatography-mass spectrometry. These differences could be related through a proteomic approach to the accumulation of cell wall modifying enzymes such as pectin methylesterases, pectin methylesterase inhibitors and xyloglucan endotransglucosylases/hydrolases in the extracellular matrix.

## Introduction

In temperate and boreal climates, plants are exposed to low temperatures on a seasonal basis. Temperatures below 0 °C can lead to freezing of plant tissues, in particular in exposed parts such as leaves. Ice crystallization takes place in the intercellular spaces^1^ and the difference between the chemical potentials of liquid water and ice leads to the growth of ice crystals by absorption of water from inside of the cells. Therefore, the temperature-dependent growth of extracellular ice crystals leads to cellular dehydration stress. Furthermore, expanding ice crystals may compress cells physically, potentially leading to disruption of the plasma membrane and cell death. Therefore, the extracellular space and the mechanical properties of cell walls can be considered to be important determinants of plant freezing tolerance.

Plants can transiently increase their freezing tolerance upon exposure to low, non-freezing temperatures (cold acclimation, CA). CA induces massive changes in gene expression that are in part regulated by the cold induced CBF transcription factors^2,3^, followed by accumulation of cold regulated (COR) proteins and compatible solutes such as sugars and proline^4–6^. In addition, modifications of membrane lipid composition have been reported^7,8^. In *Arabidopsis*, development of CA reaches a plateau after about seven days, when maximum freezing tolerance is attained^7^. Some plant species, such as wheat, rye and oat can increase their CA freezing tolerance further by an additional exposure to mild, non-damaging freezing temperatures^9–11^. This is referred to as sub-zero acclimation (SZA).

Freezing tolerance is also increased in *Arabidopsis* during SZA treatments at temperatures between −1 °C and −4 °C for up to seven days^12,13^. The molecular basis of SZA is largely different from CA because *CBF* and *COR* genes are up-regulated more moderately during SZA than CA^12^, while many genes that are not regulated at 4 °C are strongly induced or repressed at −3 °C^14^. In addition, the concentrations of compatible solutes are not significantly changed during SZA^12^. Therefore, SZA-induced changes are predicted to constitute novel mechanisms leading to increased plant freezing tolerance.

Transcriptome analysis of *Arabidopsis* leaves under SZA showed that cell wall synthesis and modification related genes are over-represented among up-regulated genes^14^. This is in agreement with electron microscopy results showing that oat leave cells accumulate xyloglucans in golgi vesicle and the extracellular space during SZA^11^. These results indicate that changes in the extracellular space including cell wall and apoplastic fluid might be key determinants of enhanced freezing tolerance during SZA.

However, only little is known about changes in cell wall and apoplast even during CA. Several studies demonstrated that cold acclimation leads to an increase in cell wall content, thickness and rigidity^15–21^. In addition, modification of pectins and hemicelluloses is associated with CA in oil seed rape and wheat^19,22^. Changes of the composition of the apoplastic fluid during CA have also been reported. For instance, the concentration of soluble sugars, such as fructans, in the apoplastic fluid is increased during SZA in cereals, suggesting that these sugars may prevent freezing injury to the plasma membrane^23–25^. In addition, the presence of antifreeze and ice binding proteins in the apoplastic fluid has been shown for several plant species after CA^26,27^. In general, however, only little is known about changes in the extracellular matrix during CA and SZA.

In the present study, we have investigated the effects of CA and SZA on *Arabidopsis thaliana*. Different accessions showed natural genetic diversity in the magnitude of increase in freezing tolerance during CA and SZA. In addition, we found significant changes in cell wall monosaccharide composition from GC-MS analysis and evidence for structural changes in the cell wall from ATR-FTIR spectroscopy. These results could be linked to the list of proteins that responded to CA and SZA in their abundance that was obtained from a proteomics approach.

## Results

### Freezing Tolerance of Arabidopsis Increased after CA and SZA

**Figure 1:**
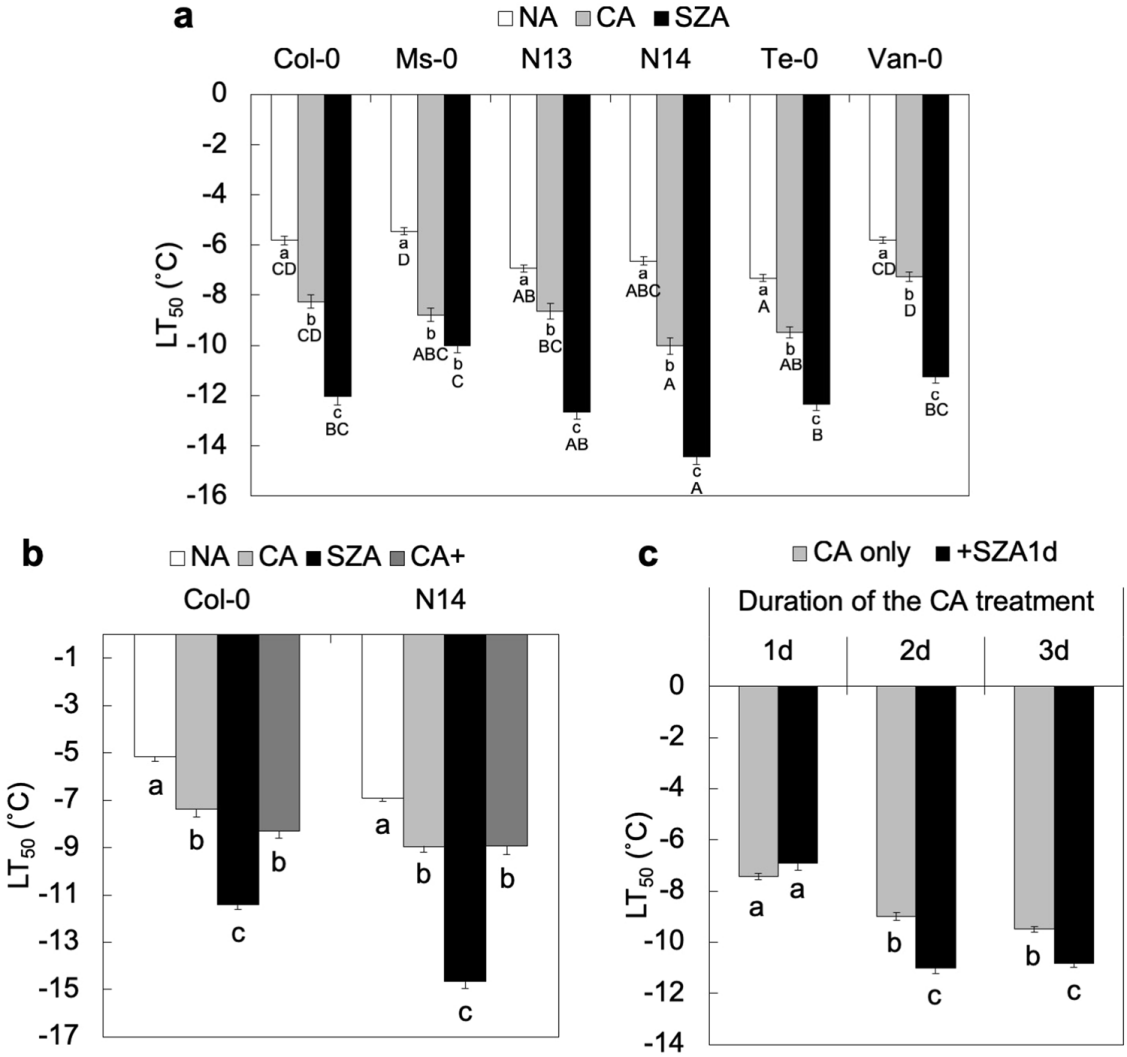
Influence of cold and sub-zero acclimation on the freezing tolerance of selected *Arabidopsis* accessions. Freezing tolerance is expressed as LT_50_ from electrolyte leakage assays. (**a**) Freezing tolerance of the accessions Col-0, Ms-0, N13, N14, Te-0 and Van-0 in the non-acclimated state (NA), after one week of cold acclimation at 4 °C (CA) and after subsequent acclimation at −3° for three days (SZA). Error bars indicate ± s.e.m. (n = 4). (**b**) Freezing tolerance (LT_50_) of Col-0 and N14 accessions under NA, CA, SZA and CA+ (extension of CA for three days under the same conditions as SZA) conditions. Error bars indicate ± s.e.m. (n = 5). (**c**) Dependence of the effect of SZA treatment (one day at −3 °C) on the duration of the CA treatment (one, two or three days) in Col-0. Error bars indicate means ± s.e.m. (n = 5) and significant differences at *p* < 0.05 (Tukey-Kramer test) between the treatments within an accession are marked with lower case letters above the bars. Significant differences in NA, CA and SZA LT_50_ among accessions are indicated by capital letters.

We first evaluated the SZA response of six *Arabidopsis* accessions that were already known to possess high freezing tolerance after CA^28^ using a well-established electrolyte leakage assay (Fig. 1a). The LT_50_ of the accessions varied by 1.87 °C, ranging from −5.45 °C (Ms-0) to −7.32 °C (Te-0) in the NA state and by 2.75 °C with a range from −7.27 °C (Van-0) to −10.02 °C (N14) after seven days of CA at 4 °C. After an additional SZA for three days at −3 °C LT_50_ varied by 4.45 °C from −10.00 °C (Ms-0) to −14.45 °C (N14). It is interesting to note that natural variation in freezing tolerance was higher after SZA than after CA. Among the six accessions, Ms-0 increased its freezing tolerance only by 1.22 °C during SZA, while N14 exhibited a more strongly enhanced freezing tolerance by 4.43 °C. For further detailed analysis we selected Col-0 as the standard accession with a good SZA response (increase in freezing tolerance of 3.80 °C) and N14 as the accession with the highest increase in freezing tolerance during SZA. This resulted in strong SZA responses in both investigated accessions, however, since they did not strongly differ in their SZA response, we did not expect major differences between the molecular reactions of the two genotypes. With this strategy, we aimed to identify robust SZA responses in *Arabidopsis*.

For the SZA treatment, *Arabidopsis* rosettes were detached from soil-grown plants and incubated in water at −3 °C in the dark. Therefore, treatment effects other than temperature could influence results obtained under SZA conditions. To control for these factors, we subjected samples to an extended cold acclimation (CA+) treatment under the same conditions as the SZA treatment, but at 4 °C. The corresponding electrolyte leakage assays with Col-0 and N14 reproduced the effects of CA and SZA treatments shown in Fig. 1a and also revealed that the CA+ treated rosettes had the same freezing tolerance as CA samples in both accessions (Fig. 1b). These results clearly show that the sub-zero temperature was the factor responsible for the increased freezing tolerance observed after the SZA treatment.

Finally, we wanted to know whether the CA treatment was required prior to SZA to trigger increased freezing tolerance. Figure 1c shows that *Arabidopsis* leaves (Col-0) did not respond to SZA with increased freezing tolerance after one day of CA at 4 °C, while after two or three days of CA this effect was clearly visible. This indicates that at least a short CA treatment is required to enable the plants to respond to the sub-zero temperature stimulus.

### Quantity and Composition of Cell Wall Material Changed during CA and SZA

**Figure 2:**
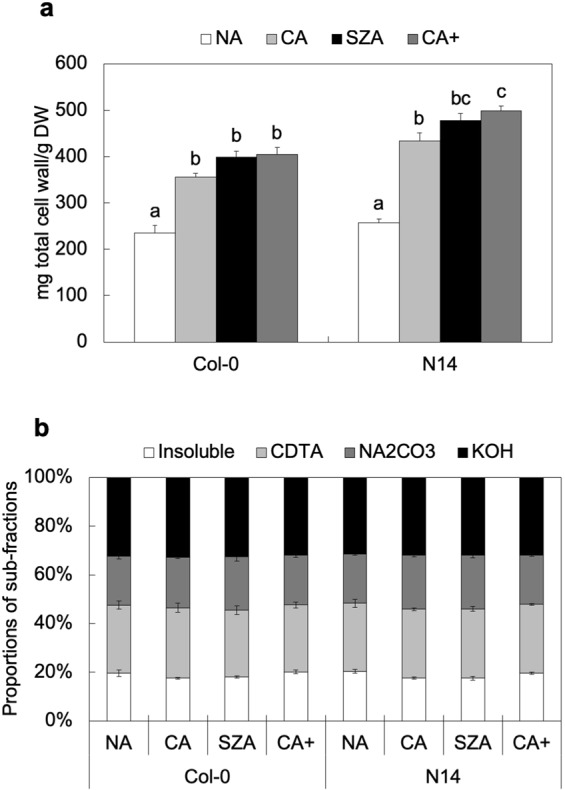
Quantitative and compositional characterization of cell walls of Col-0 and N14 under different acclimation conditions. (**a**) Amount of total cell wall material under NA, CA, SZA and CA+ conditions in Col-0 and N14 relative to total rosette dry weight (DW). Error bars indicate ± s.e.m. (n = 4) and significant differences between treatments within an accession at *p* < 0.05 (Tukey-Kramer test) are marked with different letters above the bars. (**b**) Total cell wall material was separated into four different subfractions (Insoluble, CDTA-, NA_2_CO_3_- and KOH-soluble fractions). Proportional contributions of these subfractions to the total cell wall material are shown for the different acclimation treatments.

Total cell wall material was extracted from whole rosettes of the accessions Col-0 and N14 and quantified (Fig. 2a). CA induced a significant increase of cell wall amounts in both accessions. On the other hand, after subsequent SZA and CA+ treatments, no further significant changes of cell wall content were observed, except for a small increase in N14 after CA+. Sequential extraction of total cell wall material was used to separate pectin (1,2-diaminocyclohexane tetraacetic acid (CDTA) and Na_2_CO_3_ fractions), hemicellulose (KOH fraction) and an insoluble residue containing mainly cellulose (Supplementary Fig. S1)^29–31^. Although a quantitative analysis of these four subfractions indicated that the absolute amount of each fraction increased after CA in the same way as total cell wall (data not shown), the proportions of these four fractions were maintained in the rosettes of both accessions under all conditions (Fig. 2b).

Total cell wall material and the four subfractions were used to determine the content of crystalline cellulose and uronic acid (Supplementary Figs S2–S4). As expected, cellulose was highly enriched in the insoluble fraction in both Col-0 and N14 (Supplementary Fig. S3). Similarly, uronic acid, derived from the major pectin components galacturonic and glucuronic acid^32–34^, was enriched in the CDTA and Na_2_CO_3_ fractions (Supplementary Fig. S4). While these data clearly show the successful cell wall fractionation, no significant differences were observed between SZA and CA+ in both cellulose content of the insoluble fraction and uronic acid content in the CDTA and Na_2_CO_3_ fractions. No clear CA specific changes were evident in our data except that uronic acid content was slightly higher in CA than in NA in the KOH fraction of N14.

**Figure 3:**
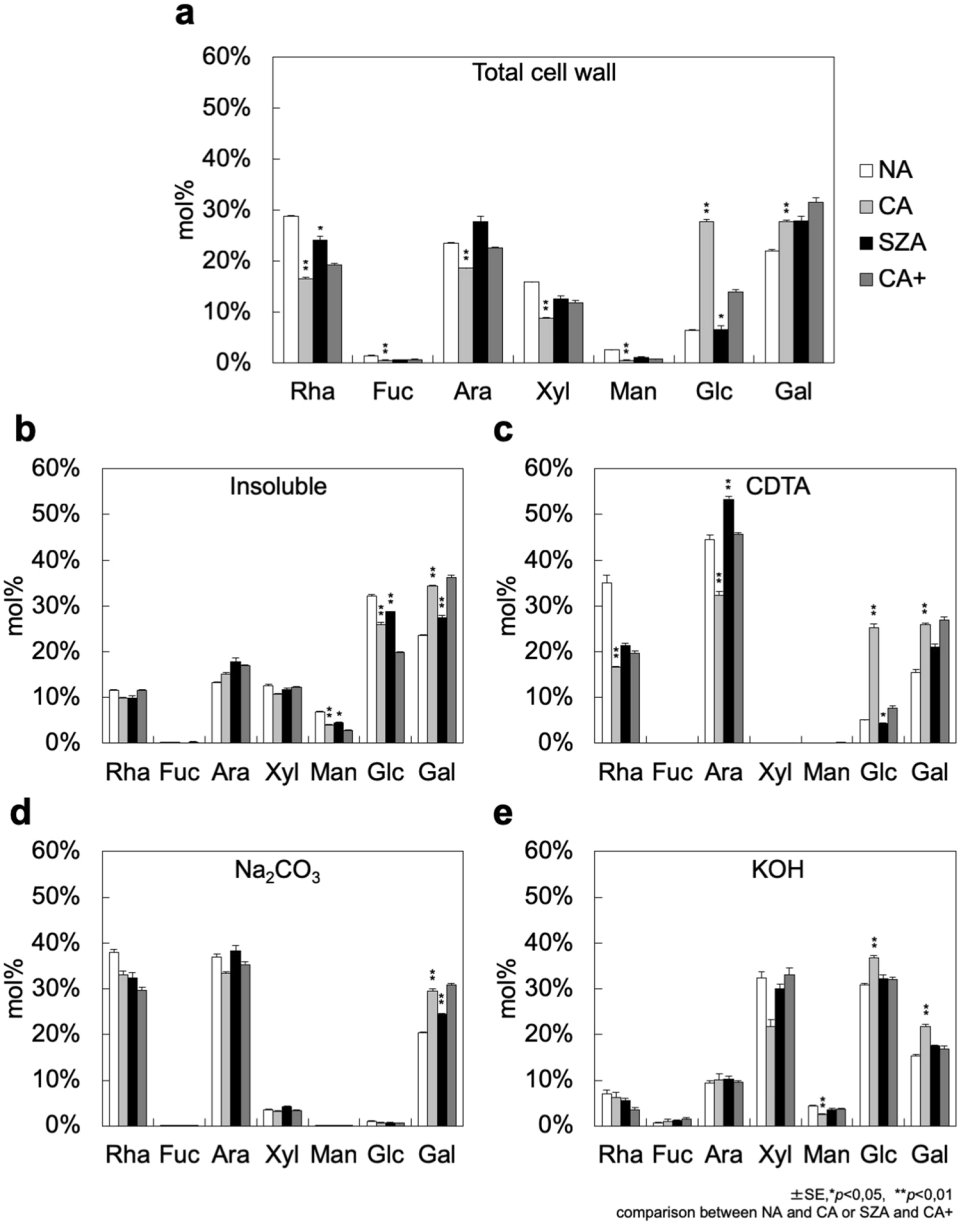
Monosaccharide composition of cell wall material isolated from Col-0 under NA, CA, SZA and CA+ conditions. Monosaccharide composition of total cell wall material (**a**) and the insoluble (**b**), CDTA-soluble (**c**), NA_2_CO_3_-soluble (**d**) and KOH-soluble (**e**) cell wall sub-fractions was characterized by GC-MS. The contribution of the single sugars is expressed as mol% of the total amount of all seven monosaccharides in each fraction. Error bars indicate ± s.e.m. (n = 3–4). Significant differences (Student’s *t*-test) between NA and CA or SZA and CA+ samples are indicated by asterisks above the bars of CA or SZA samples, respectively (*p < 0.05, **p < 0.01). The monosaccharide composition of cell wall fractions isolated from N14 is shown in Supplementary Fig. S5.

Monosaccharides were extracted from total cell wall material and the sub-fractions by trifluoroacetic acid (TFA) hydrolysis and subjected to GC-MS analysis. In the total cell wall material CA resulted in significant decreases in Rha, Fuc, Ara, Xyl and Man and increases in Glc and Gal content in Col-0 (Fig. 3a). The same changes were also observed in N14, except for Fuc (Supplementary Fig. S5). In both accessions this was due to a corresponding increase in Glc content in the CDTA fractions highly enriched in pectin. In the same fractions we also observed a significant reduction in Ara content during CA, while Rha content was reduced in the CDTA fractions extracted from all cold treated rosettes (CA, CA+, SZA). Levels of all monosaccharides were similar under CA and CA+ conditions. However, to make a stringent identification of significant changes during SZA, statistical comparisons were only made to the CA+ condition. In the total cell wall material, Rha content was significantly higher in both accessions and Glc content lower after SZA than after CA+ (Figs 3 and S5). Rha content was also slightly increased after SZA compared to CA+ in the KOH fractions, highly enriched in hemicellulose, of both Col-0 and N14. On the other hand, Glc content was slightly increased during SZA compared to CA+ in the insoluble fractions of both accessions.

### Structural Changes in the Cell Wall during CA and SZA

**Figure 4:**
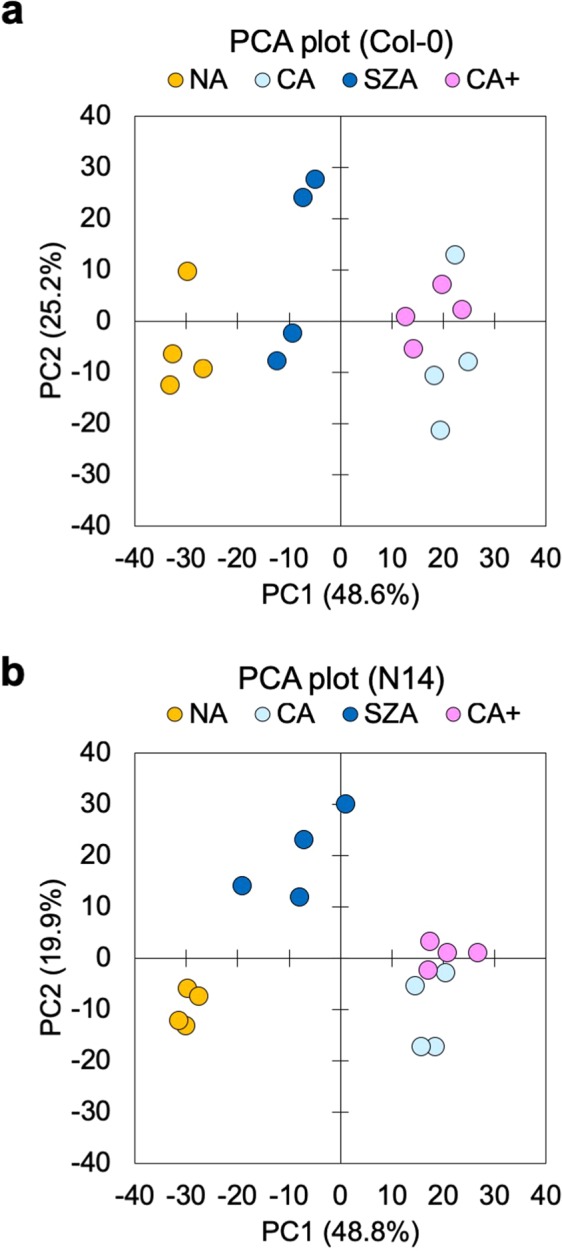
Principal component analysis (PCA) of FTIR spectra obtained from total cell wall fractions of Col-0 and N14. Dry total cell wall material was subjected to ATR-FTIR spectroscopy and spectra were further analysed by PCA. Score plots are shown for PC1 and PC2 for samples from Col-0 (**a**) and N14 (**b**). The fraction of the total variance explained by each PC is indicated in parenthesis.

The data reported above indicate that while CA lead to a massive increase in total cell wall amount, the compositional changes during both CA and SZA were rather modest. However, further structural changes leading to altered properties may occur, involving for example changes in glycosidic linkages or in methyl esterification. We therefore performed ATR-FTIR analyses on total cell wall material isolated from Col-0 and N14. The spectral range from 1800-850 cm^−1^ was used for PCA. Scores plots of spectra obtained from Col-0 (Fig. 4a) and N14 (Fig. 4b) showed three clusters of samples separated by PC1. In both cases, CA and CA+ samples formed one cluster that was clearly separated from NA samples. On the other hand, the SZA samples were located between these two clusters, but were also at least partially separated from them by PC2, indicating a unique structural character of the cell walls after SZA.

**Figure 5:**
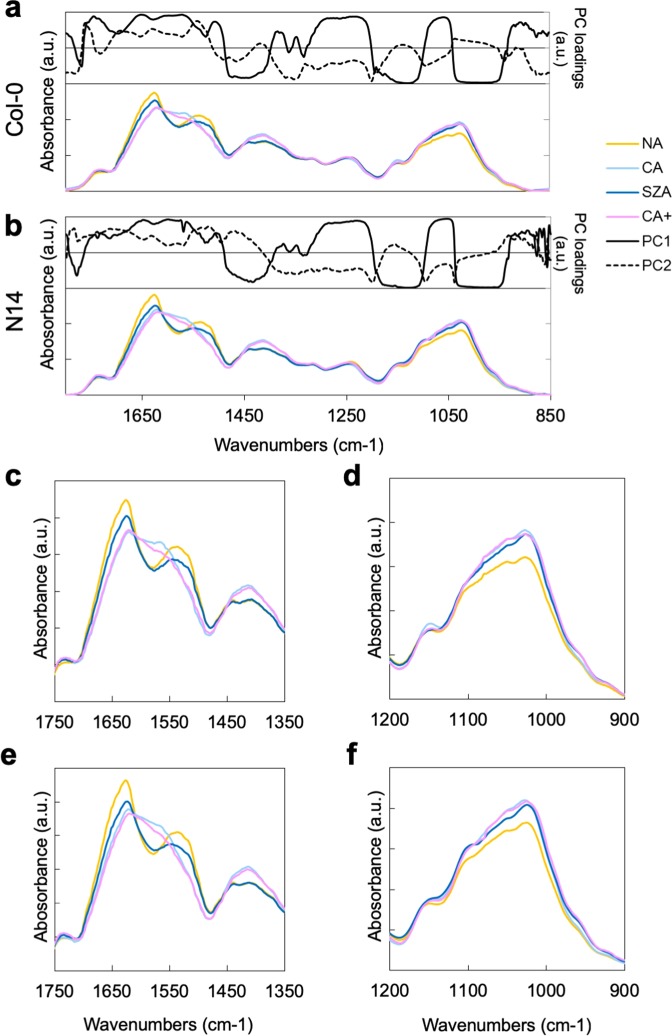
FTIR spectra of total cell wall material isolated from plants under NA, CA, SZA and CA+ conditions. The fingerprint region (1800 − 850 cm^−1^) of ATR-FTIR spectra is displayed for total cell wall material from Col-0 (**a**) and N14 (**b**). The top panels show the PC1 and PC2 loadings of the PCAs shown in Fig. 4 at each wave number. The bottom panels show the corresponding ATR-FTIR absorbance spectra of NA, CA, SZA and CA+ samples. Spectral ranges corresponding to specific cell wall structures are also indicated in these panels. (**c**–**f**) Selected spectral regions that showed differences among treatments in Col-0 (**c**,**d**) and N14 (**e**,**f**). Each spectrum is the mean of four replicate samples.

The loadings underlying PC1 and PC2 indicate the regions of the spectra that make the biggest contributions to the variance in the datasets. Plotting the loadings above the spectra revealed spectral regions that were strongly influenced by the treatments in both Col-0 and N14 (Fig. 5a,b). Of particular interest are the regions between 1750 and 1350 cm^−1^, and between 1200 and 900 cm^−1^, as they showed either strong positive or negative loadings, in particular for PC1. It has been shown that hemicellulosic polysaccharides are related to absorbance peaks in the region around 1150 cm^−1^. Likewise, the esterification of pectic polysaccharides can result in spectral changes in the regions around 1745, 1600 and 1450 cm^−1^ ^35,36^. Spectral patterns were highly similar between Col-0 and N14. In the region between 1200 and 900 cm^−1^ the spectra clearly differed between NA samples and all cold treatments (Fig. 5d,f). However, in the region between 1750 and 1350 cm^−1^, spectra from cell wall samples after SZA either showed features that were intermediate between NA and CA/CA+ samples, or that were more similar to the NA samples than to the CA/CA+ samples (Fig. 5c,e). We were not able to identify features that were specific to the SZA condition in our ATR-FTIR spectra.

### The Apoplastic Proteome was Modified during CA and SZA

The amount of cell wall material was drastically increased during CA (Fig. 2), while composition and structure were changed during both CA and SZA (Figs 3, 4 and S5). In addition, many genes encoding enzymes involved in cell wall modification are up-regulated during SZA^14^. We therefore identified proteins in the extracellular space that may be responsible for the observed cell wall changes. For this purpose, apoplastic fluids were sequentially extracted from rosettes of Col-0 and N14 using first an isotonic sorbitol solution without and subsequently one with 200 mM CaCl_2_. Soluble apoplastic proteins can be extracted with sorbitol solutions, while cell wall attached proteins are solubilized by the addition of CaCl_2_ to the solution^37^. The extracted proteins were then subjected to label-free quantitative proteomics. We were able to identify a total of 235 (Col-0) and 215 (N14) soluble apoplastic proteins from samples without CaCl_2_ and 395 (Col-0) and 376 (N14) cell wall associated proteins from samples extracted in the presence of CaCl_2_. The soluble apoplastic proteins accounted for about 15–30% of all identified proteins extracted without CaCl_2_, indicating a strong contamination in particular with plastidic proteins, while cell wall associated proteins accounted for around 70–80% of the total identified proteins in the fractions extracted in the presence of CaCl_2_ (Supplementary Table S1).

**Figure 6:**
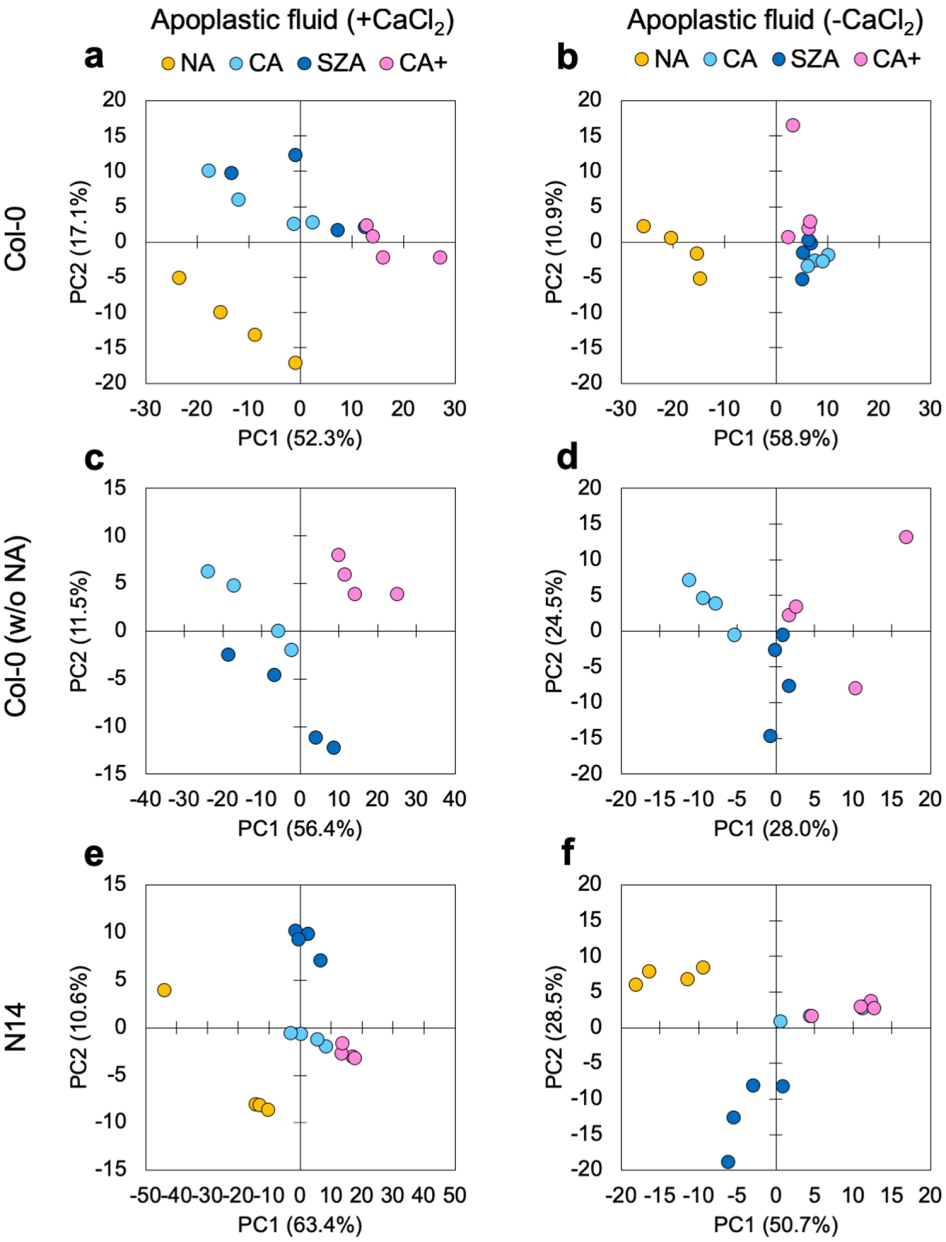
Principal component analysis of the composition of proteomes obtained from apoplastic fluids of NA, CA, CA+ and SZA treated rosettes. All apoplastic proteins identified after extraction with (**a**,**c**,**e**) or without CaCl_2_ (**b**,**d**,**f**) were included in this analysis. Scatter plots were generated with PC1 and PC2 scores in Col-0 (**a**–**d**) and N14 (**e**,**f**). Panels (c,d) were generated with the same data as (**a**,**b**), but leaving out data for the NA samples to increase resolution among the remaining treatments. The proportion of the total variance in the data sets explained by each PC is indicated. In panel (f) only one of the four CA data points is visible, because the other three are hidden by the CA+ data points.

We performed PCAs with the data sets from both accessions to obtain an overview of changes in the apoplastic proteome during CA, SZA and CA+ (Fig. 6). The analyses showed a strong separation between NA samples and all cold treated samples, regardless of the presence or absence of CaCl_2_ in the extraction buffer. In N14, CA and NA samples were clearly separated by PC1, while CA and CA+ clustered closely together (Fig. 6e,f). However, the SZA samples were separated from the CA and CA+ samples along PC2. The much lower proportion of the total variance in the data explained by PC2 than by PC1 indicates that the compositional differences between the apoplastic proteomes of CA and CA+ samples compared to the SZA samples were much smaller than the differences between any of the cold treated samples and the NA samples. In Col-0, the NA samples were also clearly separated from all cold treated samples by PC1 (Fig. 6a,b), while CA and CA+ samples were not separated from the SZA samples. When PCA was carried out without the NA samples, the SZA samples were separated from both the CA and CA+ samples (Fig. 6c,d), suggesting that the SZA treatment also induced specific apoplastic proteome responses in Col-0, similar to N14, but most likely not as extensive in the less freezing tolerant accession.

**Figure 7:**
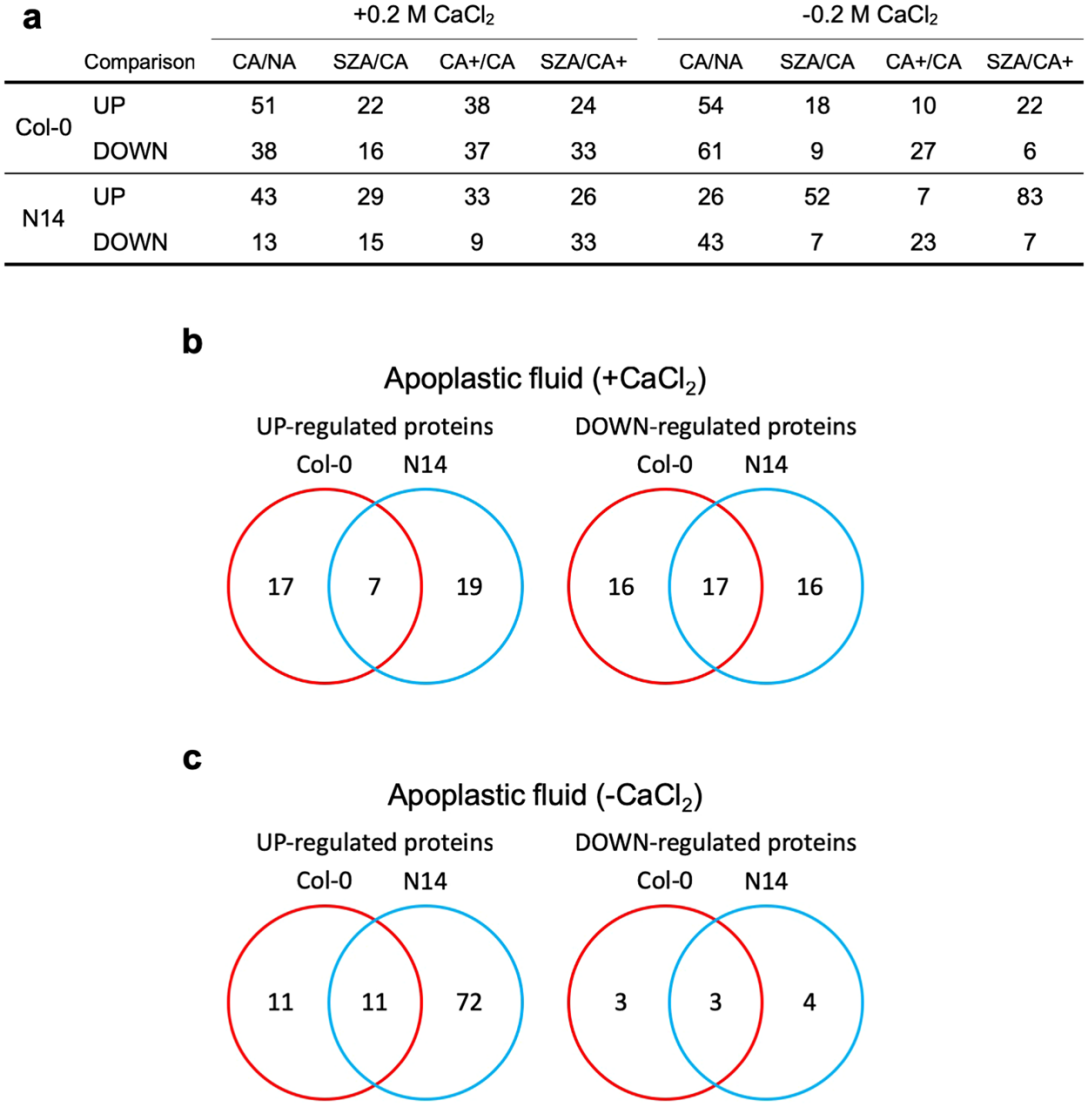
Overview of changes in the extracellular proteome of Arabidopsis leaves during CA, CA+ and SZA. (**a**) Numbers of differentially abundant proteins in apoplastic fluid extracted in the presence or absence of 0.2 M CaCl_2_. UP/DOWN refers to proteins whose abundance was significantly (*p* < 0.05) higher/lower in the indicated comparisons with a fold change >2.0 or <0.5, respectively. The overlap of proteins in the apoplastic fluid extracted with (**b**) or without CaCl_2_ (**c**) between Col-0 and N14 that were identified as being significantly differentially abundant between leaves after SZA compared to CA+ are displayed as Venn diagrams.

The number of proteins extracted in the presence of CaCl_2_ that showed significant differential abundance between treatments was similar between Col-0 and N14 (Fig. 7a). However, a large part of the proteins with significantly different abundance between SZA and CA+ were accession-specific (Fig. 7b). Apoplastic proteins extracted without CaCl_2_ showed a higher number of significant changes in abundance between CA and NA in Col-0 than in N14. On the other hand, more changes were observed between SZA and CA+ in N14 than in Col-0 for proteins with higher abundance after SZA (Fig. 7a,c) in agreement with the PCAs (Fig. 6).

Table 1 and Supplementary Table S2 provide an overview of the functions of the top 10 apoplastic proteins with the largest differences in abundance between SZA and CA+ in Col-0 and N14, respectively. Enzymes with a potential cell wall modification activity comprise a significant part of both tables. For instance, pectin methylesterases (PMEs, At1g11580 in Col-0, At3g14310 (PME3) in N14), endo-β-mannase (At5g01930), peroxidases (At4g21960 and At4g30170 in Col-0, At5g58390 in N14), xyloglucan endotransglucosylase/hydrolase (XTH22, At5g57560) and glycosyl hydrolases (At5g42720 and At3g19620 in Col-0, At4g02290 and At1g78060 in N14) were commonly increased in abundance during SZA in both accessions. Similarly, PME inhibitors (PMEIs; At2g45220 and At4g25260 (PMEI17) in Col-0, At2g45220, At3g47380 (PMEI11) and At4g25260 (PMEI17) in N14), peroxidases (At2g37130 and At5g64120 in Col-0, At2g37130, At5g39580, At4g37530 and At5g64120 in N14) and FAD-binding berberine family proteins (At1g30700 and At1g30720 in Col-0, At1g30720, At1g30730 and At1g26380 in N14) showed decreased abundance after SZA compared to the CA+ treatment. In addition to cell wall modification proteins, many pathogenesis-related (PR) proteins including chitinases and thaumatins (e.g. At3g54420 in Col-0 and At3g04720 in N14) were changed during CA and SZA in both Col-0 and N14. In most cases, however, levels of proteins that decreased during SZA were intermediate between CA and CA+, suggesting that these proteins may have increased in abundance in response to wounding or other effects of the SZA treatment during the CA+ treatment, while the sub-zero temperature had the opposite effect.

**Table 1:** Top 10 significantly differentially abundant apoplastic proteins under SZA compared to CA+ conditions in Col-0.

		AGI code	Description	log_2_FC		
				CA/NA	SZA/NA	CA+/NA
Apoplastic fluid (+CaCl_2_)	SZA/CA+ > 2.0 *p* < 0.05	AT1G11580.1	Methylesterase PCR A (PMEPCRA)	−0.112	0.339	−1.447
		AT5G05390.1	Laccase 12 (LAC12)	0.224	0.295	−1.425
		AT4G21960.1	Peroxidase superfamily protein (PRXR1)	1.619	4.696	3.042
		AT3G25530.1	Glyoxylate reductase 1 (GLYR1)	0.422	2.458	0.975
		AT5G01930.1	Endo-beta-mannase 6 (MAN6)	−0.941	−1.251	−2.715
		AT4G30170.1	Peroxidase family protein	1.452	1.310	−0.115
		AT4G33550.1	Lipid-transfer protein	0.822	0.182	−1.199
		AT5G42720.1	Glycosyl hydrolase family 17 protein	0.025	−0.178	−1.513
		AT3G47070.1	Thylakoid soluble phosphoprotein (TSP9)	−1.345	0.185	−0.985
		AT1G78040.1	Pollen Ole e 1 allergen and extensin family protein	3.180	2.699	1.569
	SZA/CA+ <0.5 p < 0.05	AT2G45220.1	Plant invertase/pectin methylesterase inhibitor superfamily	0.766	1.452	6.108
		AT4G22470.1	Lipid transfer protein family (LTP)	1.692	2.191	6.259
		AT2G02990.1	Ribonuclease 1 (RNS1)	0.053	1.053	3.964
		AT2G37130.1	Peroxidase superfamily protein	−0.589	−0.248	2.275
		AT1G74010.1	Calcium-dependent phosphotriesterase superfamily protein	0.093	1.545	3.915
		AT1G30700.1	FAD-binding Berberine family protein	1.318	2.803	5.125
		AT5G55050.1	GDSL-like Lipase/Acylhydrolase superfamily protein	0.405	1.154	3.410
		AT1G30720.1	FAD-binding Berberine family protein	1.669	0.665	2.889
		AT4G25260.1	Plant invertase/pectin methylesterase inhibitor superfamily protein	−1.282	−0.353	1.773
		AT4G33810.1	Glycosyl hydrolase superfamily protein	−0.690	−2.320	−0.395
Apoplastic fluid (-CaCl_2_)	SZA/CA+ > 2.0 p < 0.05	AT5G57560.1	Xyloglucan endotransglucosylase/hydrolase family protein (XTH22)	−0.817	2.725	0.508
		AT5G15490.1	UDP-glucose 6-dehydrogenase family protein	3.698	3.672	1.858
		AT4G19410.1	Pectinacetylesterase family protein	−1.646	−1.162	−2.783
		AT5G42240.1	Serine carboxypeptidase-like 42 (SCPL42)	−0.460	0.703	−0.898
		AT3G29360.1	UDP-glucose 6-dehydrogenase family protein	4.062	4.179	2.627
		AT1G17100.1	SOUL heme-binding family protein	1.567	2.222	0.830
		AT5G42720.1	Glycosyl hydrolase family 17 protein	1.459	1.205	−0.157
		AT3G53980.1	Lipid transfer protein family (LTP)	−0.062	−0.435	−1.730
		AT3G19620.1	Glycosyl hydrolase family protein	−1.308	−1.001	−2.220
		AT4G28780.1	GDSL-like Lipase/Acylhydrolase superfamily protein	2.882	2.510	1.313
	SZA/CA+ < 0.5 p < 0.05	AT4G22470.1	Lipid transfer protein family (LTP)	1.572	3.580	7.427
		AT2G02990.1	Ribonuclease 1 (RNS1)	1.174	1.765	5.415
		AT4G25260.1	Plant invertase/pectin methylesterase inhibitor superfamily protein	−0.510	0.847	2.815
		AT5G64120.1	Peroxidase superfamily protein	0.618	1.642	3.553
		AT3G54420.1	Chitinase class IV	0.058	0.806	2.106

Significantly more or less abundant apoplastic proteins were defined as SZA/CA+ > 2.0 and SZA/CA+ < 0.5, respectively, with p < 0.05 in both cases. All listed proteins were identified with at least two unique peptides. Numerical values show quantitative changes of protein abundances expressed as log2-fold change compared to NA samples.

## Discussion

Since relatively large amounts of plant material (around 3 g) were needed for each cell wall or apoplastic protein isolation, we modified the previously used SZA protocol, where detached leaves were incubated in glass tubes at −3 °C standing with the petioles in water. In the present study we incubated whole rosettes that were submerged in water. To ascertain that the rosettes still showed the previously observed increase in freezing tolerance, we performed electrolyte leakage assays with six *Arabidopsis* accessions and confirmed the effectiveness of our SZA treatment. A similar increase in freezing tolerance after SZA as reported here has previously been determined for the accessions Col-0 and Te-0 with detached leaves^12,14^. In addition, exposure of the rosettes to an extended CA treatment (CA+) showed that the increase in freezing tolerance during SZA was indeed due to the sub-zero temperature and not to extended time or any other experimental parameters. Significantly, we also showed that SZA requires a minimum of two preceding days of CA to result in increased freezing tolerance. This aspect will be discussed in more detail below.

In general, our results indicate that major changes in cell wall amount and composition took place during seven days of CA, while no further changes were observed when rosettes were detached and incubated in water in darkness for an additional three days at 4 °C. Three days of SZA treatment under these conditions resulted in much smaller modifications of cell wall composition than seven days of CA.

Total cell wall content was drastically increased only during CA. Such an increase in response to low temperature has previously been reported for oilseed rape, pea and a bromeliad (*Nidularium minutum*)^16,19,38^. This has been interpreted as an increase in the mechanical strength of cell walls against deformation during extracellular ice crystallization^18^. Plant primary cell walls are mainly composed of cellulose, hemicellulose and pectin. There were no changes in the cellulose or pectin content relative to total cell wall dry weight after any of the cold treatments. However, pectin accumulation during CA has been reported previously^16,19,38^, while our data showed that the relative amounts of pectin-enriched fractions (CDTA and Na_2_CO_3_) and relative uronic acid content in these fractions did not change during any of the treatments. However, the amount of pectin relative to plant dry matter also increased substantially during CA in our study, in parallel with the increase in total cell wall material.

Pectin is composed of three different components: homogalacturonan (HG), rhamnogalacturonan I (RG-I) and smaller amounts of rhamnogalacturonan II (RG-II)^39^. HG can be cross-linked by Ca^2+^ after demethylation, which results in a rigidified state referred to as pectate^40^. The total amount of pectin and the degree of HG methylation affect the pore size of the cell wall and correlate with tensile strength and the freezing tolerance of leaf tissue^18,20^. Our ATR-FTIR spectra showed differences between treatments in absorbance peaks between 1700 and 1400 cm^−1^, indicative of the degree of methylation of pectins^36^. These results indicate that pectin is methylated during CA and CA+ treatments and partially demethylated during SZA. Recently, an enhanced degree of methyl esterification has also been observed during long-term CA of winter wheat crown tissues^22^. Since the FTIR spectra give no indication of the specific sites of methylation and demethylation, the effect of SZA is not necessarily a reversion of the CA effect, but may target different sites on the pectin molecules leading to SZA-specific methylation patterns. In combination with Ca^2+^-induced cell wall stiffening and polygalacturonase-mediated cell wall softening^41^ these methylation patterns may lead to mechanical cell wall properties that support stepwise increased freezing tolerance during CA and SZA.

Mechanistically, pectin can be demethylated by PMEs and their activity can be inhibited by PMEIs^40,42^. Both PMEs and PMEIs are encoded by large gene families in *Arabidopsis* (67 *PME*, 69 *PMEI*) of which only a small number has been functionally characterized, mainly for their roles in pathogen defence^42^. In line with the FTIR results, some PMEs significantly increased in abundance during SZA and no significantly decreased PMEs were detected in either Col-0 or N14. In parallel, nine PMEIs decreased in abundance during SZA, while six low-abundant PMEIs increased in Col-0 and/or N14. In agreement with these findings, total PME activity increases in *Arabidopsis* after exposure to 0 °C^43^. In addition, overexpression of the *PMEI13* (At5g62360) gene in *Arabidopsis* decreases freezing tolerance of NA plants^42^.

RG-I is the second major pectin component. It is composed of a rhamnose/galacturonic acid backbone with arabinan, galactan or arabinogalactan sidechains attached to the backbone^39^. We found a significant increase in arabinose content in the CDTA fraction after SZA, but not after CA or CA+. The arabinose sidechains in RG-I are thought to be important determinants of the mechanical properties of cell walls, for example by preventing Ca^2+^-mediated HG cross-linking and cell wall rigidification^39^. The resurrection plant *Myrothamnus flabellifolius* has a high content of arabinose in its cell walls^44^ and the same has been found for the desiccation tolerant seeds of *Arabidopsis* and common bean (*Phaseolus vulgaris* L.)^45,46^. Moore *et al*.^47^ hypothesized that arabinose sidechains may inhibit excessive cross-linking of pectin backbones and thereby stabilize cell walls during dehydration. As extracellular ice crystallization also leads to dehydration stress, the increased arabinose content of pectin during SZA might prevent the formation of irreversible connections between pectin backbones during extracellular freezing, thereby promoting recovery of cell wall properties after thawing.

In the cell wall-bound protein fraction (+CaCl_2_), some hemicellulose-associated proteins increased specifically in abundance either during CA or SZA. For example, in both accessions XTH31 and XTH33 significantly increased specifically during CA. XTH31 has xyloglucan endohydrolase (XEH) activity *in vivo* and may regulate xyloglucan endotransglucosidase (XET) activity together with XTH17^48–50^. *XTH33* is induced by aphid infestation^51^. Overexpression of *XTH33* causes bigger leaves, wider stems and altered architecture of the adaxial epidermis^52^, suggesting that this enzyme is a major player in hemicellulose modification. Therefore, alterations of XEH and/or XET activities of XTH31 and XTH33 during CA will affect cell wall properties and contribute to enhanced freezing tolerance. On the other hand, XTH22 specifically accumulated during SZA in both Col-0 and N14. The encoding gene has been found to be cold induced in several studies^53–57^. The recombinant enzyme produced in *E*. *coli* has higher activity under cold conditions than other XTHs^58^, while the baculovirus-produced XTH22 behaves similar to other enzymes of this family^59^. XET activity of XTH22 is regulated by disulfide bond formation and/or N-linked glycosylation^60^, suggesting that low temperature conditions may cause such modifications. A recent study also revealed the presence of XET activity in XTH22^61^, suggesting that covalent cross-linking between xyloglucan chains may also play a role during SZA. In addition, the increased abundance of endo-β-mannase 6, together with various glycosyl hydrolases, peroxidases and lipid transfer proteins during SZA indicates additional cell wall modifications that will require further research to elucidate their functional relevance.

In apoplastic fractions extracted from both Col-0 and N14, many PR proteins including chitinases and thaumatins were identified. Some of these proteins were shown to be anti-freeze proteins (AFPs) in several plant species, which inhibit ice crystallization and recrystalization^26,62^. In fact, an AFP from *Lollium perenne* confers freezing tolerance when expressed in *Arabidopsis*^63,64^. PR4 protein, which significantly increased in abundance during SZA, also confers freezing tolerance in *Arabidopsis*^65^. On the whole, PR proteins identified in the present study were increased during CA, not SZA. Accumulation of these proteins during CA may modify extracellular ice formation to enhance freezing tolerance.

When NA plants were directly exposed to mild freezing temperatures without previous CA, or after only one day of CA, exposure to −3 °C did not result in increased freezing tolerance, suggesting that at least some of the changes induced during CA at 4 °C may be important for the induction of increased freezing tolerance during SZA, even if the corresponding cell wall monosaccharides, proteins and cell wall structures show no further changes during SZA. In line with this argument, a greater number of apoplastic proteins was affected by CA than by SZA. However, it is presently not clear which of the numerous molecular and structural adaptations taking place during CA are functionally relevant for a subsequent SZA treatment. Further mutational studies will be necessary to elucidate such factors.

## Methods

### Plant Cultivation and Determination of Freezing Tolerance

*Arabidopsis thaliana* plants from the accessions Col-0, Ms-0, N13, N14, Te-0 and Van-0 were grown in soil in a greenhouse at a light intensity of at least 200 μmol m^−2^ s^−1^ (8 h day length) and a temperature of 20 °C during the day and 18 °C during the night for three weeks and then at 16 h day length under the same conditions until 35 days after sowing (non-acclimated plants, NA)^66^. For cold acclimation (CA) plants were transferred to a growth chamber at 4 °C with a 16 h photoperiod (90 μmol m^−2^ s^−1^) for an additional seven days as described previously^67,68^. For sub-zero acclimation (SZA) and extended cold acclimation (CA+), whole rosettes from CA plants were transferred into 500 mL glass vials containing 20 mL distilled water und placed in a temperature controlled silicon oil bath (Huber, Offenburg, Germany) at −3 °C (SZA) or 4 °C (CA+) for three days in darkness. During the entire incubation at −3 °C, samples were supercooled under these conditions. Only for the experiment reported in Fig. 1c plants were transferred to 4 °C after 28 days of growth under non-acclimated conditions. After one, two, or three days of cold acclimation, detached leaves were exposed to one day of SZA as described previously^12^.

Leaf freezing tolerance was determined as the LT_50_ using an electrolyte leakage assay as described in detail recently^69^.

### Isolation and Fractionation of Cell Wall Material

Cell wall material (CWM) was obtained in accordance with Ruprecht *et al*.^70^, Neumetzler *et al*.^36^ and Pettolino *et al*.^71^. First, approximately 1.5–3.0 g fresh rosette material was dried at 85 °C for 3 d. After weighing, 10 mL 80% (v/v) ethanol were added and samples were homogenized in a ball mill (Retsch, Haan, Germany). Subsequently, another 10 mL 80% (v/v) ethanol were added, samples were centrifuged for 5 min at 1,500 g and supernatants were discarded. Pellets were washed at least five times with 10 mL 80% (v/v) ethanol, once with 10 mL acetone and once with 10 mL methanol. Extracted cell wall powders were air-dried. To digest starch in the cell wall preparations, 3 mL 10 mM Tris-maleate buffer (pH 6.9) was added and samples were placed in boiling water for 5 min. Starch was removed by α-amylase digestion (1 U/mg of CWM) for 1 h at 40 °C. This step was repeated with half the amount of α-amylase. Then, 12 mL of cold ethanol was added to the samples and they were incubated at −20 °C for 1 h or overnight. After centrifugation for 5 min at 1,500 g, supernatants were discarded. Pellets were washed three times with ethanol and then air-dried.

Approximately 30 mg of CWM was transferred into a screw-capped microtube for sequential cell wall fractionation (Supplementary Fig. S1). First, samples were incubated with 1.5 mL 50 mM 1,2-diaminocyclohexane tetraacetic acid (CDTA, adjusted to pH 6.5 with NaOH) at 4 °C for 12 h and centrifuged for 30 min at 14,000 g. Supernatants were collected in fresh 15 mL tubes. This was repeated twice and the supernatants combined. Samples were then sequentially extracted with 50 mM Na_2_CO_3_ and 4 M KOH (each supplemented with 20 mM NaBH_4_) in the same way. The final pellets were washed three times with distilled water. Na_2_CO_3_ and KOH fractions were acidified to pH 5.0 with acetic acid (19 μL and 900 μL, respectively). All four fractions were transferred into Spectra/Por dialysis tubes (MWCO: 3.5 kDa, Spectrum Laboratories, Rancho Dominguez, CA, USA) and dialyzed for three days at 4 °C against distilled water, which was exchanged every 12 h. All fractions were dried by lyophilisation (Christ, Osterode, Germany).

Around 1 mg of all five fractions (CWM, CDTA, Na_2_CO_3,_ KOH and insoluble) was transferred into screw-capped microtubes. An aliquot (30 μL) of 1 mg/mL *myo*-inositol was added to each sample as internal standard for GC-MS analysis. Four biological replicates of each treatment (NA, CA, SZA and CA+) were prepared.

### Determination of Crystalline Cellulose and Uronic Acids

All fractions were hydrolyzed in 250 μL 2 M trifluoroacetic acid (TFA) for 1 h at 121 °C followed by adding 300 μL 2-propanol and evaporation at 40 °C. This process was repeated twice. Then distilled water (300 μL) was added to each sample and supernatants were collected by centrifugation for 10 min at 14,000 g. Cellulose-derived hexoses were quantified with an anthrone assay^72^ and uronic acid using the m-hydroxybiphenyl/H_2_SO_4_ method^73^. Corresponding pellets were subjected to Seaman hydrolysis with 72% sulphuric acid for 1 h at room temperature^74^.

### GC-MS Analysis of Monosaccharides

Neutral monosaccharide composition of all CWM fractions was determined by gas chromatography-mass spectrometry (GC-MS). Aliquots (100 μL) of supernatants after TFA hydrolysis as described above were dried on a heating block under N_2_ gas. Subsequently, monosaccharides were converted to alditol acetates as described^75^ which is modified from Albersheim *et al*.^76^. Detection was performed with an Agilent 6890N GC System coupled with an Agilent 5973 N Mass Selective Detector (Waldbronn, Germany).

### ATR-FTIR Spectroscopy

The FTIR spectra were obtained from total CWM powder using a PerkinElmer Spectrum GX (Waltham, MA, USA) spectrometer equipped with a Golden Gate single reflection diamond ATR system (Specac Ltd., Orpington, Kent, UK). For each sample, 64 scans were accumulated in the range of 400–4000 cm^−1^. Spectra were ATR and baseline corrected using the Spectrum 10.4.3 software (PerkinElmer). They were subsequently area-normalized between 1800 and 850 cm^−1^.

### Isolation of Apoplastic Proteins

Apoplastic fluids were extracted based on the method of Boudart *et al*.^37^ with slight modifications. Aerial parts of plants were carefully removed and washed with water. Plant rosettes (Approximately 2–3 g FW) were soaked in 0.3 M sorbitol solution under vacuum. Then rosettes were placed into syringes and centrifuged in a swinging bucket rotor at 1000 × *g* for 10 min. Exuded sorbitol solution was collected as apoplastic fluid. Subsequently, rosettes were processed with 0.3 M sorbitol solution containing 0.2 M CaCl_2_ in the same way. To reduce the volume of apoplast fluids, ultrafiltration was performed using an Amicon Ultra 0.5 centrifugal filter system (molecular weight cutoff 3,000; Millipore, Billerica, MA, USA). Protein concentration of the filtrate was measured using the Pierce BCA Protein Assay Kit (Thermo Fisher Scientific, Waltham, MA, USA). Extracted proteins were stored at −80 °C. Four biological replicates of each treatment were prepared.

### Sample Preparation and Data Acquisition for nano-LC-MS/MS Analysis

After the extraction of apoplastic proteins, 50 μg of protein per sample were solubilized with 100 µL 8 M urea in 10 mM Tris-HCl (pH 8) and loaded into Microcon-30 kDa centrifugal filter units (Millipore). Proteins were reduced with 10 mM dithiothreitol and 27 mM iodoacetamide followed by digestion with trypsin (Trypsin Gold, Mass Spectrometry Grade, Promega). The digested peptides were acidified to pH < 3.0 with 10% TFA. The peptide mixture was purified and desalted on C18 SEP-Pak columns (Teknokroma, Barcelona, Spain), which were attached to a QIAvac 24 Plus (Qiagen, Hilden, Germany) vacuum manifold. The columns were equilibrated with 1 mL 100% methanol, once with 1 mL 80% acetonitrile (ACN) and twice with 1 mL 0.1% TFA. The peptides were applied to the C18 column and allowed to pass through slowly. The column was washed twice with 1 mL of 0.1% TFA. Peptides were eluted with 800 µL 60% ACN, 0.1% TFA, dried in a vacuum concentrator and stored at −80° prior to mass spectrometry analysis.

Peptides were resuspended in 30 μL of resuspension buffer (5% v/v ACN, 2% v/v TFA). Measurements were performed on a Q Exactive Plus coupled to an Easy nLC1000 HPLC (Thermo Scientific). Eight μL of the samples were loaded onto an Acclaim PepMap RSLC reversed-phase column (75 μm inner diameter, 15 cm length, 2 µm bead size; Thermo Scientific) at a flow rate of 0.4 μL min^−1^ in a buffer consisting of 3% (v/v) ACN, 0.5% (v/v) acetic acid. Peptide elution was facilitated by increasing the ACN concentration from 3% to 30% (v/v) linearly over 46 min, from 30% to 50% over the next 12 min and from 50% to 80% for the last 10 min. The column was then washed with 80% (v/v) ACN for 7 min at a flow rate of 0.3 μL min^−1^. Peptide ions were detected in a full scan from mass-to-charge ratio 200 to 1600 at a resolution of 70,000 (AGC target of 3e6 and maximum IT of 100 ms). MS/MS scans were performed for the 15 peptides with the highest MS signal (ddMS2 resolution of 17,500, AGC target 1e5, isolation width mass-to-charge ratio 2 m/z, relative collision energy 30). Peptides for which MS/MS spectra had been recorded were excluded from further MS/MS scans for 15 s.

Quantitative analysis of MS/MS measurements was performed with Progenesis LC/MS software (Nonlinear Dynamics). Selection of a reference run, alignment and peak picking were performed automatically. The spectra for each MS1 signal peak were exported to Mascot. Mascot search parameters were set as follows: Arabidopsis TAIR10 genome annotation, requirement for tryptic ends, fixed modification: carbamidomethylation (cysteine), variable modification: oxidation (methionine), peptide mass tolerance =±10 ppm, MS/MS tolerance =±0.8 Da, allowed peptide charges of +2 and +3 and maximum of two missed cleavages. Spectra were also searched against a decoy database of the Arabidopsis proteome and results were filtered to ensure a FDR below 1% on the protein level. Additionally, peptide identifications with a Mascot score below 25 were excluded. Mascot results were imported into Progenesis QI. Relative quantitation using Hi-N was selected as a protein quantitation option. The three most abundant peptides were used for this analysis. Lists of peptides and proteins identified in the present study are shown in Supplementary Tables S3 and 4, respectively.

Apoplastic proteins were predicted by SUBA3 (http://suba.plantenergy.uwa.edu.au/) and the total sum of the apoplastic protein abundances was normalized across all samples.

### Statistical Analysis

Statistical significance of differences was determined by Student’s *t*-test for two-group comparisons and by Tukey-Kramer test for multiple comparisons at the p < 0.05 level. The data from FTIR and proteome analysis was Pareto-scaled for principal component analysis (PCA) using the *prcomp* function in the R software.

## Supplementary information

Supplementary Fig. S1, S2, S3, S4; Supplementary Table S1, S2

Supplementary Table S3

Supplementary Table S4

## Data Availability

The mass spectrometry proteomics data have been deposited in the ProteomeXchange Consortium database^77^ via the PRIDE partner repository with the dataset identifier PXD010575 and 10.6019/PXD010575. All other data are included in the supplemental files or are available from the corresponding author on reasonable request.
