# Supplementary material for: Both cold and sub-zero acclimation induce cell wall modification and changes in the extracellular proteome in Arabidopsis thaliana
Source: Sci Rep. 2019 Feb 19;9:2289. doi: 10.1038/s41598-019-38688-3 (PMC6381082; doi:10.1038/s41598-019-38688-3)

# Both cold and sub-zero acclimation induce cell wall modification and changes in the extracellular proteome in *Arabidopsis thaliana*

Daisuke Takahashi[1], Michal Gorka[1], Alexander Erban[1], Alexander Graf[1], Joachim Kopka[1], Ellen Zuther[1] and Dirk K. Hincha[1*]

[1]Max-Planck-Institut für Molekulare Pflanzenphysiologie, Am Mühlenberg 1, D-14476 Potsdam, Germany

* hincha@mpimp-golm.mpg.de

Corresponding author:

Dirk K. Hincha

Max-Planck-Institut für Molekulare Pflanzenphysiologie

Am Mühlenberg 1, Potsdam, 14476, Germany

Tel/Fax: +49-(0)331-567-8253

E-mail: hincha@mpimp-golm.mpg.de

Supplemental data legends

Supplementary Fig. S1. Diagram overview of sequential cell wall fractionation adapted from Ruprecht et al. (2011), Neumetzler et al. (2012) and Pettolino et al. (2012).

Supplementary Fig. S2. Amounts of crystalline cellulose and uronic acid in total cell wall material isolated from Col-0 and N14. (a) Proportions of crystalline cellulose in total cell wall material. (b) Proportions of uronic acid in total cell wall material. Weight % were calculated relative to total cell wall weight (A and B). Error bars indicate ±s.e.m. (n =3-4). Significant differences between treatments within an accession at p<0.05 (Tukey-Kramer test) are marked with different letters above the bars.

Supplementary Fig. S3 Amounts of crystalline cellulose in the four sub-fractions isolated from Col-0 and N14. Weight % were calculated relative to weight of the insoluble (**a**), CDTA-soluble (**b**), NA_2CO_3-soluble (**c**) and KOH-soluble (**d**) cell wall sub-fractions. Error bars indicate ±s.e.m. (n =3-4). Significant differences between treatments within an accession at $p<0.05$ (Tukey-Kramer test) are marked with different letters above the bars.

Supplementary Fig. S4. Amounts of uronic acid in the four sub-fractions isolated from Col-0 and N14. Weight % were calculated relative to weight of the insoluble (**a**), CDTA-soluble (**b**), NA_2CO_3-soluble (**c**) and KOH-soluble (**d**) cell wall sub-fractions. Error bars indicate ±s.e.m. (n =3-4). Significant differences between treatments within an accession at $p<0.05$ (Tukey-Kramer test) are marked with different letters above the bars.

Supplementary Fig. S5. Compositions of cell wall monosaccharides isolated from N14 under NA, CA, SZA and CA+ conditions. Monosaccharide composition of total cell wall material (**a**) and the insoluble (**b**), CDTA-soluble (**c**), NA_2CO_3-soluble (**d**) and KOH-soluble (**e**) cell wall sub-fractions was characterized by GC-MS. The contribution of the single sugars is expressed as mol% of the total amount of all seven monosaccharides in each fraction. Error bars indicate ±s.e.m. (n =3-4). Significant differences (Student's *t*-test) between NA and CA or SZA and CA+ samples are indicated by asterisks above the bars of CA or SZA samples, respectively (**p*<0.05, ***p*<0.01).

Supplementary Table S1. Subcellular localization of identified proteins. Potential protein location were predicted by SUBA3. Each value indicates percent of each predicted targeting site caluculated from normalized abundance of each proteins identified.

Supplementaly Table S2. Top 10 significantly differentially abundant apoplastic proteins under SZA compared to CA+ conditions in N14. Significantly more or less abundant apoplastic proteins were defined as SZA/CA > 2.0 and SZA/CA < 0.5, respectively, with p<0.05 in both cases. All listed proteins were identified with at least two unique peptides. Trend lines show quantitative changes of protein abundances expressed as log2-fold change compared to NA samples.

Supplementary Table S3. List of identified and quantified peptides in *Arabidopsis* apoplastic fluids. [a]Modification type and its location in peptide sequence identified, [b]Assigned proteins identified by MASCOT search engine, [c]Whether or not to use peptide in quantification with ProgenesisQI.

Supplementary Table S4. List of identified and quantified proteins in *Arabidopsis* apoplastic fluids. [a]All peptide assigned to individual proteins. [b]Unique peptide assigned to individual proteins. [c]Confidence score generated by Progenesis QI, [d]Potential protein location predicted by SUBA3.

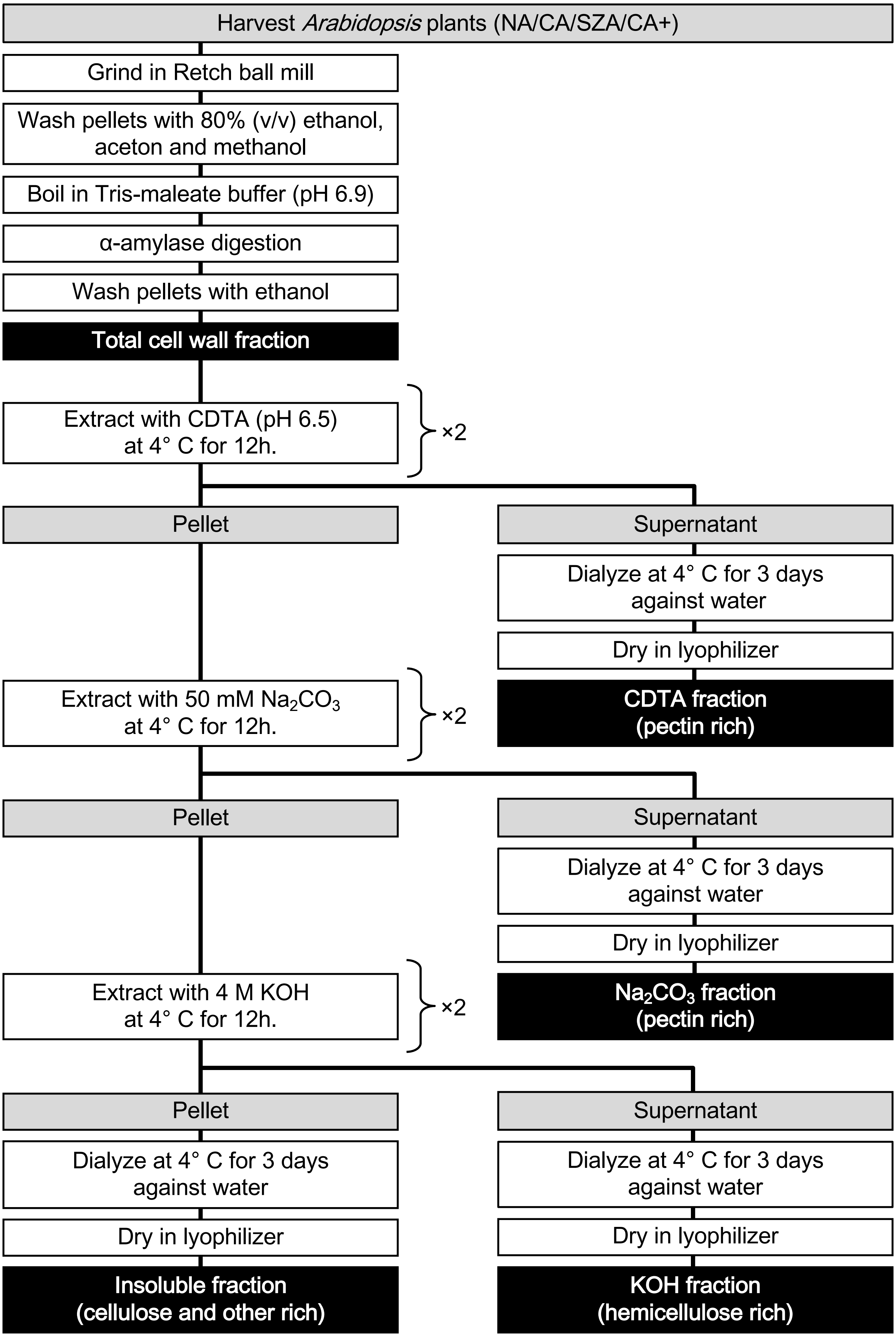

Supplementary Fig. S1.

a

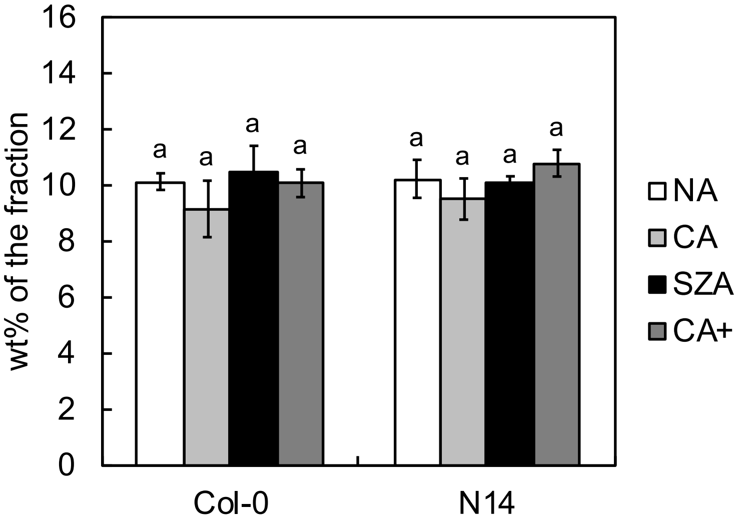

b

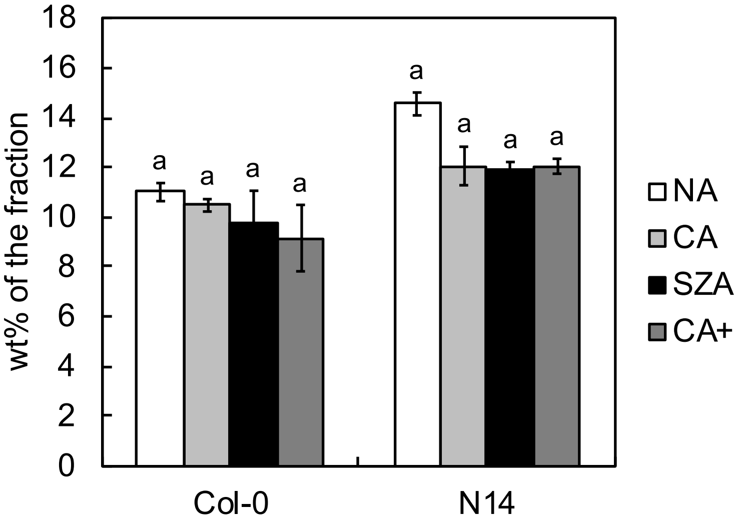

Supplementary Fig. S2.

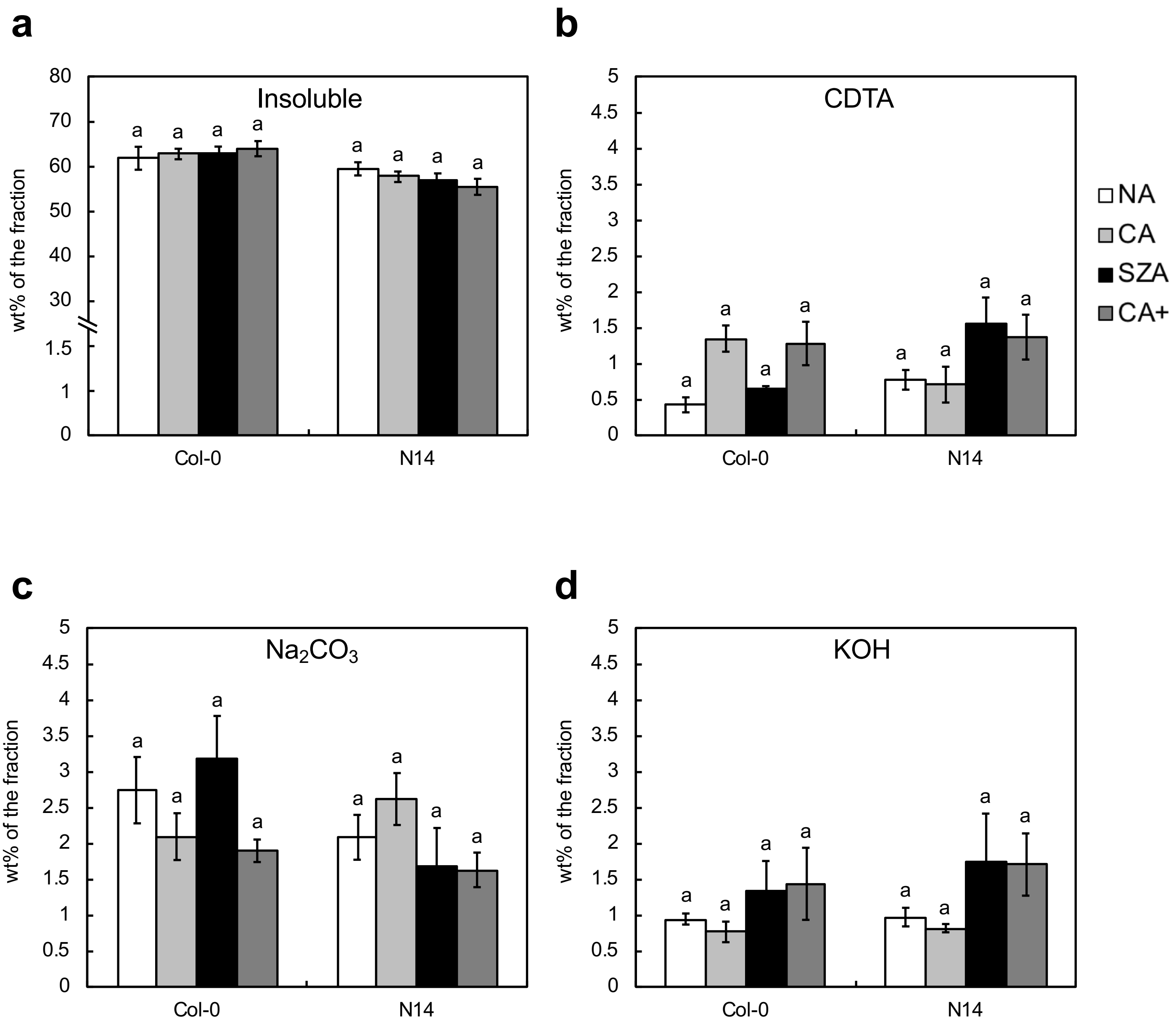

Supplementary Fig. S3.

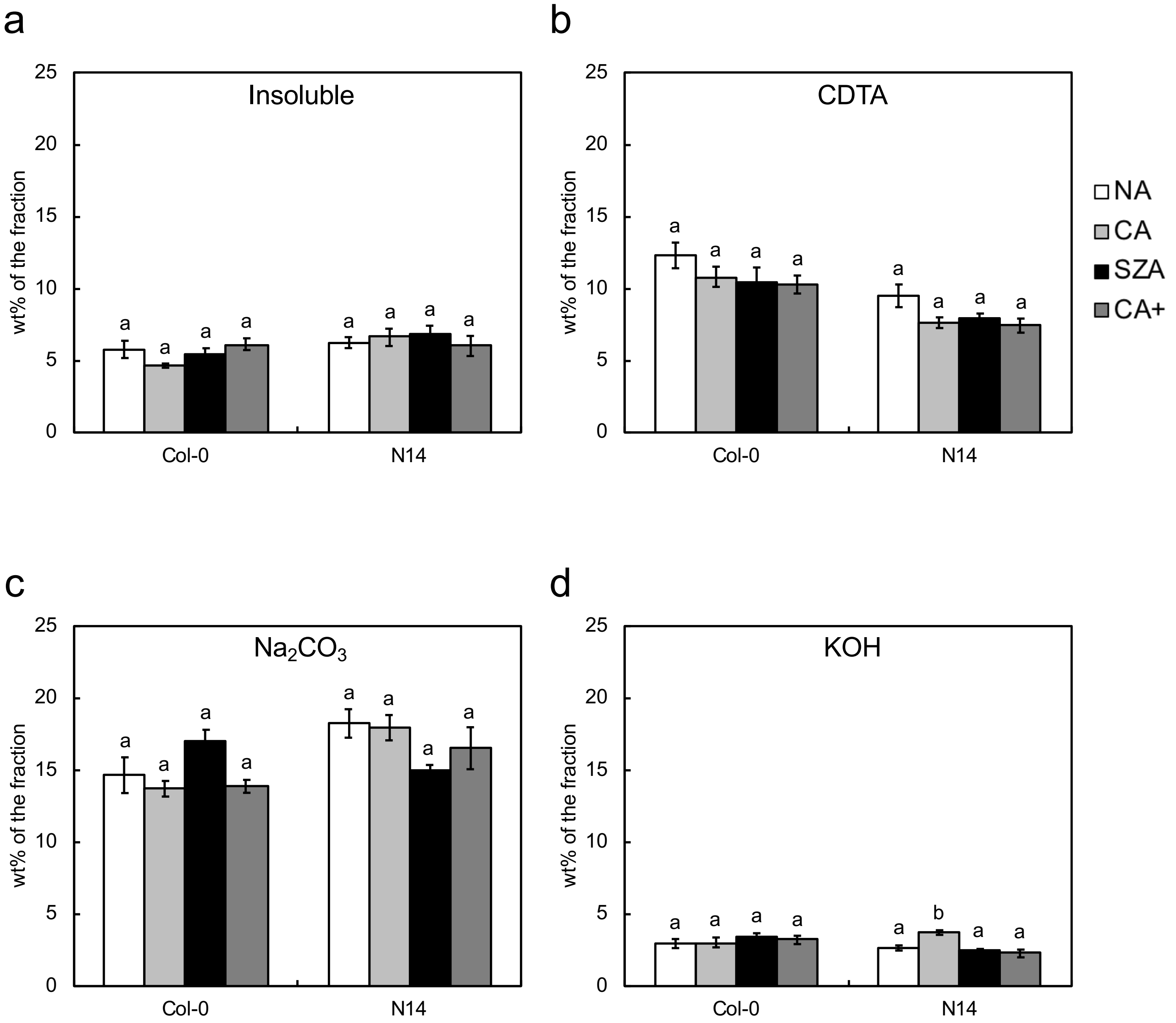

Supplementary Fig. S4.

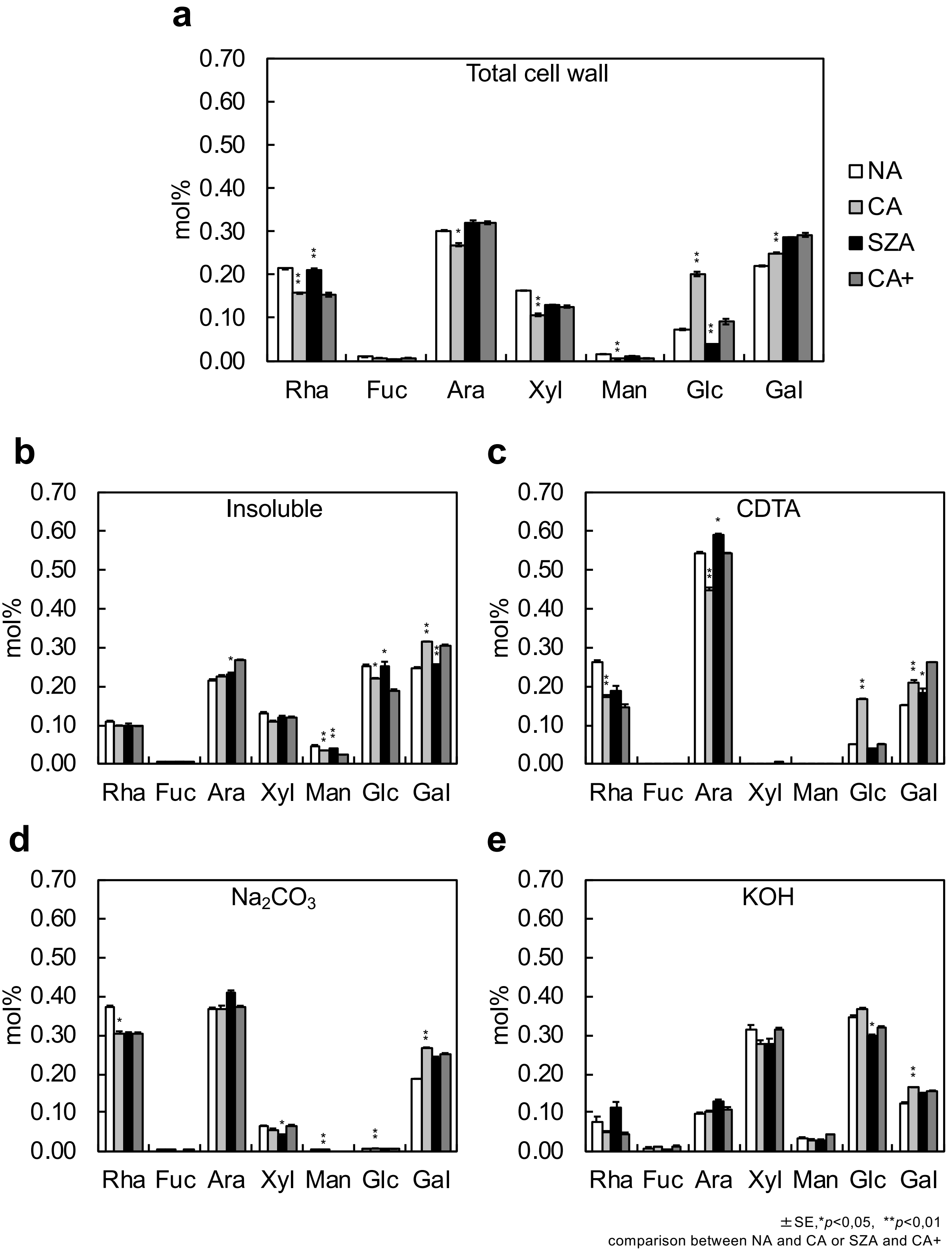

Supplementary Fig. S5.

Supplementary Table S1.

	Col-0								N14							
	Apoplastic fluid (+$CaCl_2$)				Apoplastic fluid (-$CaCl_2$)				Apoplastic fluid (+$CaCl_2$)				Apoplastic fluid (-$CaCl_2$)			
	NA	CA	SZA	CA+	NA	CA	SZA	CA+	NA	CA	SZA	CA+	NA	CA	SZA	CA+
Extracellular	76.23	77.17	71.65	74.83	24.08	14.03	13.21	17.41	77.70	77.08	68.82	79.98	15.27	21.85	19.24	29.78
Extracellular,ER	0.01	0.01	0.01	0.01	0.05	0.03	0.02	0.02	0.04	0.02	0.01	0.02	0.13	0.07	0.05	0.08
Extracellular,Plasma Membrane	0.72	0.75	0.59	0.67	0.74	0.73	0.63	0.65	0.56	0.71	0.50	0.65	0.56	0.48	0.39	0.45
Cytosol,Extracellular	4.95E-03	0.01	0.02	0.01	0.01	0.05	0.04	0.03	0.01	0.01	0.01	0.01	0.04	0.06	0.04	0.04
Mitochondrion,Extracellular	0	0	0	0	1.09E-03	1.23E-03	4.74E-03	2.06E-03	0	0	0	0	0	0	0	0
**Extracellular Total**	**76.96**	**77.94**	**72.27**	**75.53**	**24.88**	**14.84**	**13.90**	**18.11**	**78.32**	**77.82**	**69.35**	**80.67**	**15.99**	**22.46**	**19.73**	**30.35**
Cytosol	2.49	2.61	3.11	2.31	6.72	12.27	12.28	11.23	2.61	2.64	3.49	2.13	10.52	12.92	12.10	9.21
Cytosol,Mitochondrion	0	0	0	0	0.05	0.08	0.07	0.05	0.02	0.01	0.01	0.01	0.07	0.07	0.06	0.05
Cytosol,Nucleus	0.09	0.11	0.14	0.09	0.24	0.52	0.50	0.34	0.23	0.16	0.21	0.18	0.31	0.67	0.42	0.53
Cytosol,Plasma Membrane	0.04	0.07	0.09	0.07	0.20	0.43	0.45	0.42	0.05	0.08	0.11	0.06	0.25	0.41	0.44	0.40
Cytosol,Plastid	1.54E-04	5.33E-04	1.85E-03	5.32E-04	0.01	0.02	0.01	0.01	1.56E-03	1.42E-03	2.80E-03	1.02E-03	0.02	0.02	0.02	0.02
Cytosol,Plastid,Nucleus	3.24E-03	3.78E-03	0.01	2.50E-03	0	0	0	0	0	0	0	0	0	0	0	0
Cytosol,Vacuole	0	0	0	0	0.01	0.02	0.02	0.02	1.68E-03	1.35E-03	3.88E-03	1.08E-03	0.02	0.02	0.02	0.02
ER	0.07	0.09	0.14	0.08	0.14	0.27	0.30	0.28	0.09	0.13	0.17	0.11	0.14	0.25	0.29	0.24
ER,Golgi	0	0	0	0	0	0	0	0	0	0	0	0	0.01	0.02	0.02	0.01
ER,Peroxisome	0	0	0	0	0.01	0.01	0.01	0.01	0	0	0	0	1.66E-03	3.98E-03	2.46E-03	2.10E-03
Golgi	0	0	0	0	0	0	0	0	0.02	0.01	0.01	0.02	0	0	0	0
Mitochondrion	1.99	1.81	1.96	2.44	2.96	2.40	2.52	2.43	1.03	1.04	1.53	0.92	2.24	2.36	2.71	2.19
Mitochondrion,Plastid	0.01	0.01	0.02	0.01	0.06	0.08	0.07	0.04	0.01	0.02	0.02	0.01	0.06	0.07	0.07	0.07
Nucleus	0.37	0.56	0.79	0.53	0.30	0.60	0.82	0.46	0.86	0.98	1.59	1.40	0.29	0.52	0.86	0.45
Nucleus,Plasma Membrane	0.01	0.01	0.02	0.01	0.01	0.01	0.01	0.01	1.60E-03	3.97E-03	0.01	3.45E-03	2.47E-03	5.00E-03	2.45E-03	1.08E-03
Peroxisome	0.33	0.35	0.41	0.28	1.08	1.23	1.27	1.58	0.24	0.29	0.36	0.21	1.08	1.32	1.34	0.99
Plasma Membrane	1.45	1.35	1.28	1.54	1.08	0.92	0.88	0.84	5.15	1.77	1.53	2.04	0.88	0.93	0.91	1.03
Plasma Membrane,ER	9.18E-04	1.75E-03	0.01	2.10E-03	4.69E-03	0.01	0.01	3.82E-03	1.06E-03	2.99E-03	0.01	2.13E-03	4.07E-03	0.01	0.01	0.01
Plasma Membrane,Golgi	5.45E-05	4.73E-04	1.26E-03	1.04E-03	0	0	0	0	0	0	0	0	0	0	0	0
Plastid	14.96	13.54	18.25	15.39	61.69	65.67	66.18	63.22	9.98	13.19	19.96	10.03	67.32	57.04	59.95	53.41
Plastid,Peroxisome	0	0	0	0	0.02	0.02	0.02	0.02	0	0	0	0	0.01	0.02	0.02	0.01
Vacuole	1.17	1.47	1.45	1.61	0.49	0.51	0.56	0.78	1.35	1.81	1.55	2.11	0.71	0.70	0.85	0.87
Vacuole,ER	0.03	0.03	0.03	0.06	0.03	0.02	0.03	0.06	0.03	0.04	0.04	0.07	0.02	0.03	0.03	0.05
Vacuole,Golgi	0.02	0.02	0.03	0.03	0.02	0.04	0.05	0.04	0.02	0.01	0.02	0.02	0.02	0.04	0.04	0.03
Vacuole,Plasma Membrane	2.14E-03	1.52E-03	1.57E-03	9.55E-04	0.02	0.02	0.02	0.01	1.50E-03	9.52E-04	1.22E-03	9.43E-04	0.03	0.11	0.09	0.05
Total	100	100	100	100	100	100	100	100	100	100	100	100	100	100	100	100

Supplementary Table S2.

		AGI code	Description
Apoplastic fluid (+$CaCl_2$)	SZA/CA+ >2.0 *p* <0.05	AT4G02290.1	Glycosyl hydrolase 9B13 (GH9B13)
		AT5G01930.1	Endo-beta-mannase 6 (MAN6)
		AT5G58390.1	Peroxidase superfamily protein
		AT5G57560.1	Xyloglucan endotransglucosylase/hydrolase family protein (XTH22)
		AT4G00230.1	Xylem serine peptidase 1 (XPS1)
		AT3G14310.1	Pectin methylesterase 3 (PME3)
		AT3G11780.1	MD-2-related lipid recognition domain-containing protein
		AT3G14067.1	Subtilase family protein
		AT3G62020.1	Germin-like protein 10 (GLP10)
		AT3G04720.1	Pathogenesis-related 4 (PR4)
	SZA/CA+ <0.5 p<0.05	AT2G45220.1	Plant invertase/pectin methylesterase inhibitor superfamily
		AT2G37130.1	Peroxidase superfamily protein
		AT2G02990.1	Ribonuclease 1 (RNS1)
		AT3G47380.1	Plant invertase/pectin methylesterase inhibitor superfamily protein
		AT1G30720.1	FAD-binding Berberine family protein
		AT5G39580.1	Peroxidase superfamily protein
		AT3G61490.1	Pectin lyase-like superfamily protein
		AT4G37530.1	Peroxidase superfamily protein
		AT2G41800.1	Protein of unknown function, DUF642
		AT1G30730.1	FAD-binding Berberine family protein
Apoplastic fluid (-$CaCl_2$)	SZA/CA+ >2.0 p<0.05	AT3G14310.1	Pectin methylesterase 3 (PME3)
		AT3G16370.1	GDSL-like Lipase/Acylhydrolase superfamily protein
		AT1G31710.1	Copper amine oxidase family protein
		AT5G63810.1	β-galactosidase 10 (BGAL10)
		AT3G54400.1	Eukaryotic aspartyl protease family protein
		AT1G78060.1	Glycosyl hydrolase family protein
		AT4G25900.1	Galactose mutarotase-like superfamily protein
		AT1G78850.1	D-mannose binding lectin protein with Apple-like carbohydrate-binding domain
		AT2G02850.1	Plantacyanin (ARPN)
		AT3G44100.1	MD-2-related lipid recognition domain-containing protein
	SZA/CA+ <0.5 p<0.05	AT2G02990.1	Ribonuclease 1 (RNS1)
		AT4G24040.1	Trehalase 1 (TRE1)
		AT5G64120.1	Peroxidase superfamily protein
		AT1G26380.1	FAD-binding Berberine family protein
		AT4G25260.1	Plant invertase/pectin methylesterase inhibitor superfamily protein

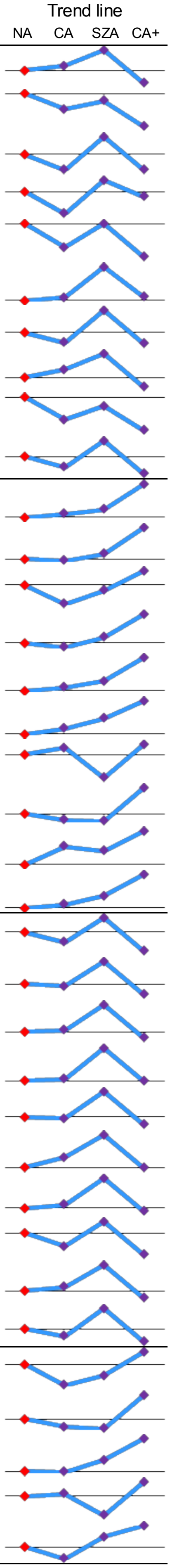

Supplement: Supplementary file 1 — Supplementary Fig. S1, S2, S3, S4; Supplementary Table S1, S2 [file 41598_2019_38688_MOESM1_ESM.pdf]
